# Radiation Effects on Brain Extracellular Matrix

**DOI:** 10.3389/fonc.2020.576701

**Published:** 2020-10-02

**Authors:** Elvira V. Grigorieva

**Affiliations:** ^1^Institute of Molecular Biology and Biophysics, Federal Research Center of Fundamental and Translational Medicine, Novosibirsk, Russia; ^2^V. Zelman Institute for Medicine and Psychology, Novosibirsk State University, Novosibirsk, Russia

**Keywords:** glioblastoma radiotherapy, brain irradiation, extracellular matrix, proteoglycan expression, chondroitin sulfate, heparan sulfate, heparanase, metalloproteinase

## Abstract

Radiotherapy is an important therapeutic approach to treating malignant tumors of different localization, including brain cancer. Glioblastoma multiforme (GBM) represents the most aggressive brain tumor, which develops relapsed disease during the 1st year after the surgical removal of the primary node, in spite of active adjuvant radiochemotherapy. More and more evidence suggests that the treatment's success might be determined by the balance of expected antitumor effects of the treatment and its non-targeted side effects on the surrounding brain tissue. Radiation-induced damage of the GBM microenvironment might create tumor-susceptible niche facilitating proliferation and invasion of the residual glioma cells and the disease relapse. Understanding of molecular mechanisms of radiation-induced changes in brain ECM might help to reconsider and improve conventional anti-glioblastoma radiotherapy, taking into account the balance between its antitumor and ECM-destructing activities. Although little is currently known about the radiation-induced changes in brain ECM, this review summarizes current knowledge about irradiation effects onto the main components of brain ECM such as proteoglycans, glycosaminoglycans, glycoproteins, and the enzymes responsible for their modification and degradation.

## Introduction

Adjuvant chemoradiotherapy with temozolomide is a conventional protocol for the standard treatment for newly diagnosed glioblastoma multiforme (GBM) (1). Last decades, new treatment approaches have shown promise in improving GBM outcomes and are described in details in the reviews (2, 3).

Radiotherapy (RT) represents an essential part of this post-surgery GBM treatment, and the current standard usually includes 30 X-ray fractions × 2 Gy, although multiple non-conventional RT sources and regimens are under active investigation (3–5).

RT aims to destroy the residual GBM cells at the resection border and prevent the disease relapse, but it has wide biological effects on different molecular and physiological parameters in the irradiated brain tissue as well (6). Negative side effects of X-ray radiation include: increased permeability of the blood-brain barrier (7, 8); brain necrosis (9); morphological changes, microvascular injury, and activation of astrocytes after irradiation of mouse brain (10); metabolic and histopathological changes in the specific rat brain regions (11); suppressed cell proliferation in the hippocampal subgranular zone (12) and long-term neurocognitive impairment (13, 14).

Along with that, RT-induced lymphodepletion and subsequent suppression of immune response contribute to the insufficient efficiency of conventional RT and may be limiting the success of GBM treatment (15). Also, irradiation affects the expression of microRNA (16) and numerous biomarkers related to inflammation, DNA damage and repair, cell activation and damage, angiogenesis pathways, which are involved in the pathogenesis of GBM and its radioresponse (17, 18). Latest knowledge on radiation-induced genetic and epigenetic changes as well as a role of reactive oxygen species (ROS), GBM heterogeneity, and tumor microenvironment (TME) in brain tumor biology is presented in the comprehensive review by Raviraj et al. (19).

At present, more and more evidence is accumulating showing that TME is actively involved in the molecular fate of GBM tumor-initiating cells and tumor development, and significantly modifies the epigenetic landscape of GBM cells with unknown mechanism (19). Irradiation affects normal brain microenvironment, resulting in changes in hippocampal neurogenesis and attenuates tolerance of normal brain after cranial irradiation (20). These results are supported by the fact that after complete resection of the tumor mass and chemoradiotherapy, GBM commonly recurs around the tumor removal site, suggesting that the microenvironment at the tumor border provides therapeutic resistance to GBM cells (21). These interactions between glioma cells and the brain microenvironment can influence glioma pathobiology and contribute to its poor prognosis (22).

The presented data demonstrate that different components of the GBM TME (including oxidative stress and inflammation, immune response, and angiogenesis) actively respond to X-ray irradiation, whereas the contribution of extracellular matrix (ECM) to radiation-induced changes in both GBM tumor and normal brain tissue remain much less investigated. The available information on this issue is very scanty and fragmentary, but it may be useful to summarize it in order to outline possible directions for future research in this scientific field.

Here, the effects of experimental and clinical irradiation onto key brain ECM components (such as proteoglycans, glycosaminoglycans, glycoproteins, and their modifying enzymes) in normal brain tissue and GBM cells/tumors will be reviewed.

## Proteoglycans

Proteoglycans (PGs) are the main components of brain ECM and play important roles in normal brain physiology and gliomagenesis (23). They represent core proteins with covalently-attached polysaccharide chains of glycosaminoglycans (GAGs), which are responsible for cell-cell and cell-matrix communication and signaling and related to the formation of a permissive provisional matrix for tumor growth (24, 25). PGs are both involved in primary glioma development and contribute to therapeutic resistance of cancer stem cells (CSCs) and GBM relapse development (26). Irradiation-induced changes in PGs composition, content, or localization can be one of the potential molecular mechanisms related to accelerated proliferation and invasion of GBM cells and the disease progression.

### Chondroitin Sulfate Proteoglycans

A major component of the brain ECM is chondroitin sulfate proteoglycans (CSPGs), which are actively involved in the organization of GBM TME and glioma invasion (27). The complex distribution of CSPGs in the tumor microenvironment can determine the invasion potential of glioma cells through the coordination of ECM-cell adhesion and dynamic changes in stromal cells (28). Among the main brain CSPGs are neurocan, brevican, CSPG4/NG2, CD44, aggrecan, versican, decorin, and biglycan.

**Figure 1:**
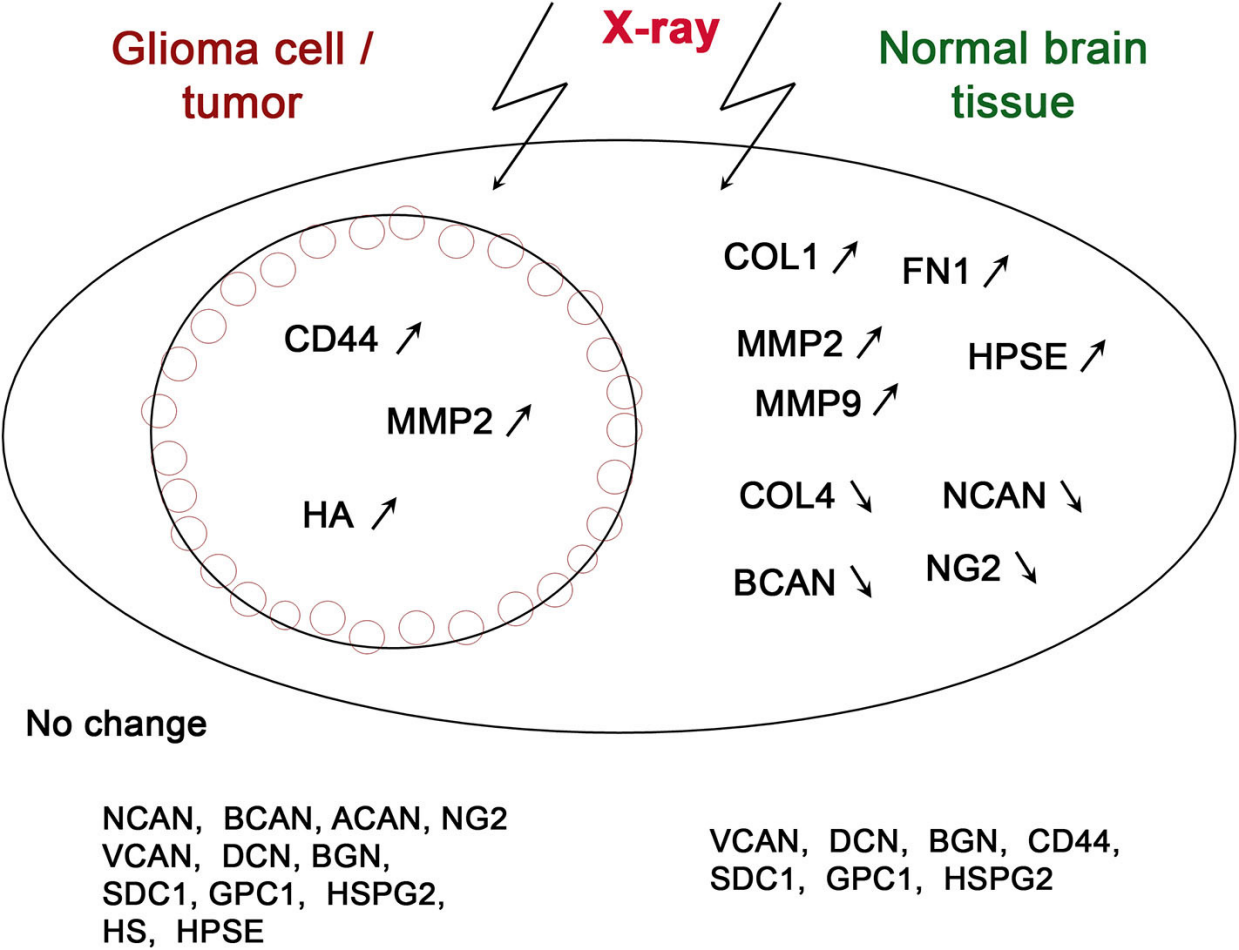
Schematic illustration of irradiation-induced changes in the expression of various ECM components in glioma cells/tumors and normal brain tissue. The genes, which expression changes were shown at least in a single study, are depicted on the scheme. Arrows correspond to up-regulation (↗) or down-regulation (↘) of the gene expression.

The few available data on radiation effects on CSPGs are quite controversial, although expression of CSPG-coding genes has a tendency to be down-regulated in non-tumor tissues and up-regulated in glioma cells or tissues upon X-ray irradiation (Table 1, Figure 1). Cranial radiotherapy of patients with small-cell lung cancer with a dose of 60–80 Gy significantly decreases brevican and neurocan content in cerebrospinal fluid at 3- and 12-months' time-points after irradiation (34). Expression profiling of main CSPG core proteins in normal mouse brain upon single X-ray irradiation with a dose of 7 Gy demonstrates quick (24–72 h) decrease of brevican and neuro-glial antigen-2 (NG2/CSPG4) mRNA levels (3–10 and 8–9-fold, respectively), whereas expression of neurocan, versican, CD44, decorin, and biglycan was not affected (30). As neuro-glial antigen2 (NG2/CSPG4) is a specific biomarker of oligodendrocyte progenitor cells (OPCs) and glioma cancer stem cells (CSC) (26), indirect data on the changes in NG2 expression upon irradiation could be deducted from the changes of OPC cells, which are characterized by high NG2 content. X-irradiation of the adult rat spinal cord decreases the number of OPCs at 4 and 10 days after irradiation (41). According to Irvine and Blakemore, irradiation results in almost complete depletion of OPCs within the telencephalon (cortex, corpus callosum, and hippocampus) by 7 days post-irradiation, which was followed by progressive repopulation of OPCs from non-irradiated areas of the cortex. By contrast, within the lower brain centers (the diencephalon and mesencephalon), the OPC loss occurred much more slowly so that 26% of the OPCs still remained 4 weeks after X-irradiation (42). These results suggest that OPCs may induce stemness and chemo-radioresistance in GBM cells and participate in the formation of a “border niche,” a unique microenvironment that allows GBM cells to survive and recur at the tumor border (21).

**Table 1:** Irradiation effects onto brain ECM components.

		**Irradiation zone**	**Irradiation type**	**Regimen dose, Gy**	**Detection after**	**Analyzed**	**Methods**	**ECM components**	**Non-ECM parameters**	**References**
**Normal brain tissue**										
1.	Fisher 344-Brown Norway male rats	Brain	^137^Cs γ-irradiator	Single, 10 Gy	4, 8, 24 h	Brain tissue	IHC, RT-PCR zymography	Collagen IV (↘), MMP2 (↗), MMP9 (↗)	TIMP-1, TIMP-2	(29)
2.	C57BL/6 male mice	Brain	^137^Cs γ-irradiator	8 × 5 Gy, twice a week	4, 8, 24 h	Brain tissue	IHC, RT-PCR zymography	Collagen IV (↘), MMP2 (↗)	TIMP-2	(29)
3.	CBL/6Bl male mice	Brain	Synchrotron and clinical linear accelerators ElektaAxess	Single, 7 Gy	24, 48, 72 h	Brain tissue	RT-PCR	Syndecan-1 (=), Glypican-1 (=), Perlecan (=), Versican (=), CD44 (=), Brevican (↘), Neurocan (=), NG2/CSPG4(↘), Decorin (=), Biglycan (=)		(30)
4.	Mouse brain microvessel endothelial cells	Cell culture	^137^Cs γ-irradiator	4.4–5.7 Gy	6, 24 h	Cells	Cell morphologyzymography	Heparanase (↗)	Mitogenic factors	(31)
5.	Rhesus macaques male	Brain	Clinical linear accelerator	8 × 5 Gy, twice a week	6 months	Brain tissue	RT-PCR	Fibronectin I (↗), MMP2 (↗)	SERPINE1, VEGFB, TNFSF15, SYP, CCL2, GRIA4, CD68, GRIN2A	(32)
6.	Rhesus macaques male	Brain	Clinical linear accelerator	8 × 5 Gy, twice a week	6 months	Brain tissue	IHC	Fibronectin I (↗), Collagen I (↗), Collagen IV (=), MMP9 (↗)	Rho/ROCK, RAAS signaling pathways	(33)
7.	SCLC patients	Brain	Clinical linear accelerator	15 × 2 Gy or 20 × 4 Gy	3 months, 12 months	Cerebrospinal fluid	ELISA	Brevican (↘), Neurocan (↘)	Chemokine, cytokine and proinflammatory panels	(34)
**Glioma cells and tumors**										
8.	U251 cells	Cell culture	Clinical linear accelerator Clinac 600 C/D	2, 5, 10, and 20 Gy		Cells	MTS test	GBM cell survival		(35)
9.	Nude-nu female mice	Orthotopic U251 brain tumor	Implanted ^125^I seeds	0.2 mCi for 26 days	26 days	Brain tissue	IHC	NG2/CSPG4	PRDX-1, ATM, Chk2, ASK, pH2AX	(35)
10.	U87MG, U251 cells	Cell culture	^137^Cs (Gammacell 1,000 unit)	2, 4, and 6 Gy	24 h	Cells	WB zymography	MMP2, (↗) in U87 cells, (=) in U251 cells	TIMP2, p53	(36)
11.	U87 xenografts in nude mice	Orthotopic U87 brain tumor	Linear accelerator CLINAC 21EX	Single, 6 Gy or 5 × 2 Gy	11–16 days	Tumor tissue	IHC zymography	MMP2 (↗)	P53, GFAP	(36)
12.	U87 cells	Cell culture	^137^Cs γ-irradiator	2 Gy/day for 3 days		Cells, conditioned medium	RT-PCR ICC, IHC, ELISA, Western blot	Brevican (=), Versican (=), Neurocan (=), Aggrecan (=), CD44 (↗), HA (↗), COL1A1 (=), COL3A1 (=)	HAS1 (–), HAS2 (+), HAS3 (–), VIM, CDH-2, NF-kB, cytokines	(37)
13.	U87 xenografts in mice	Brain	137Cs X-rays source	2.5 Gy/day for 3 days		Tumor tissue	IHC	CD44 (↗), HA (↗)	HAS2	(37)
14.	Human GBM tissues	Glioma	Clinical accelerator			Tumor tissue	IHC	CD44 (↗), HA (↗)	HAS2, VIM, ZEB1	(37)
15.	Primary human GBM cells xenografts in nude rats	Orthotopic tumor area	Collimated dorso-ventral beam of 6 MV X-rays	Single, 50 Gy	7 weeks	Brain tissue	IHC	CD44 (↗), MMP2 (↗)	Ki-67, SDF-1a, HIF-1a, vWF	(38)
16.	U87 and U251 cells	Cell culture	X-rays accelerator	2 Gy	1–13 h	Cells	qRT-PCR	Circ_VCAN (↘)	miR-1183	(39)
17.	Glioma patients					Glioma tissue	qRT-PCR	Circular RNA from versican (Circ_VCAN)	miR-1183	(39)
18.	Human GBM tissues	Glioma relapse tumor	Clinical linear accelerator	30 × 2 Gy	6–12 months	Tumor tissue	IHC	HS (=), Heparanase (=)		(40)

*Arrows correspond to up-regulation (↗) or down-regulation (↘) of the gene expression, =, no change in the expression level upon irradiation*.

In contrast with normal brain tissue, experimental irradiation of the U251 orthotopic tumors in the brain of Nude-nu female mice by 0.2 mCi iodine-125 (^125^I) seed results in an increase of NG2 content in the brain tissue. The U251 cells with activated NG2 expression (U251–NG2) were significantly more resistant to 5 Gy irradiation compared to the NG2-negative U251 cells suggesting NG2 as an important prognostic factor for radiotherapy resistance (35). X-ray irradiation of U87MG GBM cells (^137^Cs τ-rays source, 2 Gy/day for 3 days) activates the expression of CD44 in these cells (37), and CD44 protein content was elevated in primary human GBM tumors that were developed in nude rat brain and undergone irradiation at a dose of 50 Gy, by 4 weeks after irradiation (38). At the same time, expression levels of brevican, neurocan, aggrecan, and versican in irradiated U87MG cells was not changed (37). Although a significant decrease in the expression of circular noncoding RNA from versican (circ_VCAN, hsa_circ_0073237) in irradiated U87 and U251 cells by 13 h post-irradiation was demonstrated (4–5- and 8–9-fold, respectively), it was significantly up-regulated in radioresistant glioma tissues compared with the radiosensitive tissues. Both irradiation and knockdown of circ_VCAN inhibit proliferation, migration, and invasion of the cells, while overexpression of circ_VCAN promotes migration and invasion of the irradiated glioma cells (39).

As to the contribution of polysaccharide CS chains in radiation sensitivity, there are no data up-to-date.

Overall, the presented data suggest brevican and neurocan as sensitive targets for X-ray radiation, while high expression levels of NG2 and versican impart radioresistance to glioma cells, and all the CSPGs contribute to the irradiation-induced ECM reorganization during GBM radiotherapy.

### Hyaluronic Acid

Hyaluronic acid (HA) is an integral component of any tissue, but it plays a particularly important role in the brain, where it represents a major component of intercellular space (43).

Irradiation effects onto HA remain to be almost uninvestigated. The only study by Yoo et al. demonstrates that irradiation of GBM U87MG cells (2 Gy/day for 3 days) or U87MG orthotopic brain tumors (2.5 Gy/day for 3 days) results in a significant increase of HA content in GBM cell-conditioned medium and experimental tumors, respectively. The increase of HA content in tumor tissue affects the biomechanical tension in the GBM microenvironment and provides pro-invasive extracellular signaling cue due to binding with CD44 receptor and SRC activation sufficient for a mesenchymal shift of GBM cells. These findings suggest an explanation for the frequent brain tumor relapse after radiotherapy (37).

### Heparan Sulfate Proteoglycans

Heparan sulfate proteoglycans (HSPGs) represent one of the main components of the neurogenic niche. Tight involvement of both the polysaccharide heparan sulfate (HS) chains and their degrading enzyme heparanase in the development of the nervous system and growth and invasion of glioma tumors are comprehensively described by Xiong et al. (44, 45). Among the main brain HSPGs are syndecan (1-4) and glypican (1-6) families, perlecan/HSPG2, agrin, and collagen XVIII.

There is just fragmentary information on the irradiation effects onto these macromolecules.

Experimental irradiation of normal mouse brain (7 Gy in a single dose) did not change expression levels of syndecan-1, glypican-1, and perlecan in short-term period (24–72 h) (30). However, HSPGs content is deteriorated in the brain tissue of GBM patients: high expression of glypican-1 in GBM patients who received adjuvant radiochemotherapy significantly correlates with their survival and predicts poor prognosis (46). At the same time, immunohistochemical analysis for polysaccharide chains of HS in primary and post-radiochemotherapy relapsed GBM tumors from the same patients (matched pairs) did not reveal significant changes in HS content (40).

## Glycoproteins

Despite the common extracellular glycoproteins (fibronectin, collagens, laminin, and elastin) not being much abundant in brain tissue, they are intrinsic components of brain ECM. Radiation-induced changes in their expression contribute to pathological brain ECM reorganization during radiotherapy.

Whole-brain irradiation of experimental animals (a single dose of 10 Gy for rats or 8 fractions × 5 Gy for 4 weeks for mice) decreases collagen IV content in brain tissue by 24 h after irradiation, while no significant changes are shown at 4 or 8 h after irradiation. Simultaneous up-regulation of expression and enzymatic activity of MMP2 and MMP9 seems to be a molecular mechanism for irradiation-induces ECM degradation (29).

Long-term effects (6 months) of irradiation of the whole brain of Rhesus macaques (age 6–11 years), which had received 40 Gy (8 fractions of 5 Gy each, twice per week), increased expression of fibronectin 1 (FN1) in brain tissue at mRNA level (32) and protein content of fibronectin and collagen I (COL1), whereas collagen IV (COL4) protein level was not affected (33).

Irradiation of U87 GBM cells (2 Gy/day for 3 days) does not affect the expression of collagen type-I alpha 1 (COL1A1) and collagen type III alpha 1 (COL3A1) (37).

## ECM Modifying Enzymes

Brain ECM structure is tightly determined by a complex interplay between the expression of ECM glycoproteins/PGs/GAGs and their proteolytic remodeling by matrix metalloproteinases (MMPs) and GAG-degrading enzymes (47, 48). Radiation affects both the expression/deposition of ECM components and activation/repression of the ECM-modifying enzymes, directly contributing to the overall ECM remodeling upon irradiation (Table 1, Figure 1).

### Metalloproteinases

X-ray irradiation of animal brain results in a quick (4–24 h) up-regulation of expression and enzymatic activity of MMPs: MMP2 and MMP9 (for rats, single dose) and MMP2 (for mice, 8 × 5 Gy, twice a week) (39). Irradiation-induced changes in MMPs expression seem to possess long-term effect—brain tissues of Rhesus macaques irradiated at a similar regimen (8 × 5 Gy, twice a week) demonstrate significantly increased mRNA level for MMP2 (32) and MMP9 protein content (33) at 6 months after irradiation.

Irradiation of U87MG GBM cells at 2–6 Gy doses increases expression and enzymatic activity of MMP2 in these cells and experimental orthotopic U87 tumors obtained from them (36). This result perfectly corresponds to the data on irradiation effects on the primary human GBM tumors developed in nude rats described by Shankar et al. (38) Immunohistochemical staining confirmed a significant increase of MMP2 content in the irradiated experimental tumors after 4 weeks after irradiation.

### Heparanase

Heparanase (HPSE) is the main enzyme responsible for the degradation of polysaccharide chains of HS at the cell surface and ECM of all tissues. It involved in normal physiology and pathological reorganization of ECM into TME and cancer progression and metastasis (31, 45, 49).

Gamma-irradiation of brain microvessel endothelial cells results in a significant increase in the release of heparanase, which degrades [35S]-labeled heparan sulfate from the subendothelial matrix. This was most pronounced at the 24 h after irradiation and can affect the interactions of tumor cells with endothelial cells and their microenvironment, which in turn facilitate the formation of metastasis in irradiated tissues (32). The relapsed post-radiochemotherapy GBM tumors demonstrate significantly higher intertumor and intratumor heterogeneity of heparanase (HPSE) content and distribution compared with the matched primary GBM tumors from the same patient (40).

## Discussion

According to the presented data, X-ray radiation affects all key components of normal brain ECM (PGs, collagens, MMPs, and heparanase) in different extent and directions (Figure 1).

An interesting observation is that radiation has a much pronounced effect on normal brain tissue than on tumor cells. There is a common tendency to the increased expression of glycoproteins (collagen and fibronectin) and decreased expression of main proteoglycan components of brain ECM (brevican, neurocan, and NG2/CSPG4) indicating significant changes of normal structure of brain ECM (Figure 1). Replacement of negatively-charged PGs with more neutral glycoprotein molecules results in the decrease of overall negative charge, and attenuated molecular signaling and cell-cell/cell-matrix interactions. Activation of metalloproteinases (MMP2, MMP9) and heparanase (degradating protein- and polysaccharide ECM components) contributes to even more significant reorganization of brain ECM and its transformation to pro-invasive microenvironment.

On the other side, GBM cells and tumors demonstrate more resistant phenotype to X-ray irradiation and completely different pattern of radiation-induced changes. Only three of fifteen ECM components (CD44, MMP2, and hyaluronic acid) respond to irradiation by activation of their expression/content (Figure 1). The coordinated increase of hyaluronic acid and its receptor (CD44) in tumor tissue affects the biomechanical tension in the GBM microenvironment tightly related to invasive capacity of glioma cells (37).

These radiation-induced changes in brain ECM and the residual GBM cells cooperate to provide a favorable microenvironment for GBM progression. Moreover, multiple studies show that radiotherapy not only serves as a therapeutic mean to eliminate glioma cells but also activates proliferation and invasion of those cells which survived irradiation. This seems to occur due to the selection of radioresistant GBM clones and their active invasion in previously irradiated GBM tumor microenvironment (50).

Molecular mechanisms of negative side effects of irradiation are tightly related to GBM TME: subcurative irradiation of primary human GBM tumors in rat brain results in the increased proliferation (3-fold), migration, and invasion of the survived GBM cells associated with increased expression of CD44 and activation of MMP2 expression (38); irradiation increases the invasion of U87 cells and the capacity of GBM cells to contract collagen gels, indicating that radiation changes biomechanical tension (37); irradiation of U87MG cells activates invasion of these cells and increases tumor margin invasiveness in nude mice *in vivo*, possibly through the imbalance between MMP2 and TIMP2 and ECM degradation (36). Besides the direct influence on the proliferative and invasive potential of glioma cells, radiation stimulates them to secrete regulatory molecules which in turn contribute to TME reorganization. For example, conditioned medium/ECM from irradiated U87 cells was more pro-invasive compared with ECM conditioned by non-irradiated cells (37).

Radiation induces also significant changes in immune components of brain microenvironment, which contributes to poor efficacy of anti-GBM radiochemotherapy. Irradiation results in activation of the expression of immune-associated genes (especially related to the chemokine signaling and IL-6 signaling pathways) in murine glioma and human glioma U87 cell line, leading to changes in immune microenvironment, glioma cells radioresistance, and treatment failure (51). Cranial irradiation reduced CD206 expression and increased IL1-beta expression in the mouse brain associated with the absence of monocyte-derived macrophages and long-lasting inflammation (52). Irradiation-induced release of pro-inflammatory cytokines in brain tissue microglial activation might be caused by microglial activation and is mediated by the PIDD-C/NF-κβ transcription pathway (53). In common, these data reveal immune component of brain TME as potential theraupeutic targets for combined immunotherapy and radiotherapy to treat GBM patients.

Thus, GBM radiotherapy possesses a double effect—it directly affects brain ECM and induces GBM cells to modulate their microenvironment. The balance between these modalities might be responsible for the ambiguous functional effect *in vivo*, where the initial positive effect on inhibition of GBM tumor growth by 14 day is followed by the tumor relapse and higher mortality for the mice with a smaller tumor volume by 21 day (54). Overall, the presented data underline the importance of further studies of radiation effects on GBM TME and molecular mechanisms of the normal brain's tolerance to irradiation, which may provide opportunities to improve the conventional anti-glioblastoma radiotherapy and prevent GBM relapse development.

## Author Contributions

EG conceived the study, drafted, and edited the manuscript.

## Conflict of Interest

The author declares that the research was conducted in the absence of any commercial or financial relationships that could be construed as a potential conflict of interest.

## References

[1] Stupp R Hegi ME Gilbert MR Chakravarti A. Chemoradiotherapy in malignant glioma: standard of care and future directions. J Clin Oncol. (2007) 25:4127–36. 10.1200/JCO.2007.11.8554 17827463

[2] Mohtashami E Shafaei-Bajestani N Mollazadeh H Mousavi SH Jalili-Nik M Sahebkar A. The current state of potential therapeutic modalities for glioblastoma multiforme: a clinical review. Curr Drug Metab. (2020) 10.2174/1389200221666200714101038. [Epub ahead of print]. 32664839

[3] Tan AC Ashley DM López GY Malinzak M Friedman HS Khasraw M. Management of glioblastoma: state of the art future directions. CA Cancer J Clin. (2020) 70:299–312. 10.3322/caac.21613 32478924

[4] Dhermain F. Radiotherapy of high-grade gliomas: current standards and new concepts, innovations in imaging and radiotherapy, and new therapeutic approaches. Chin J Cancer. (2014) 33:16–24. 10.5732/cjc.013.10217 24384237 PMC3905086

[5] Liu EK Sulman EP Wen PY Kurz SC Novel therapies for glioblastoma. Curr Neurol Neurosci Rep. (2020) 20:19 10.1007/s11910-020-01042-6 32445058

[6] Hau E Shen H Clark C Graham PH Koh ES L McDonald K. The evolving roles and controversies of radiotherapy in the treatment of glioblastoma. J Med Radiat Sci. (2016) 63:114–23. 10.1002/jmrs.149 27350891 PMC4914819

[7] Yoshida Y Sejimo Y Kurachi M Ishizaki Y Nakano T Takahashi A. X-ray irradiation induces disruption of the blood-brain barrier with localized changes in claudin-5 and activation of microglia in the mouse brain. Neurochem Int. (2018) 119:199–206. 10.1016/j.neuint.2018.03.002 29545059

[8] Yuan H Gaber MW Boyd K Wilson CM Kiani MF Merchant TE. Effects of fractionated radiation on the brain vasculature in a murine model: blood-brain barrier permeability, astrocyte proliferation, ultrastructural changes. Int J Radiat Oncol Biol Phys. (2006) 66:860–6. 10.1016/j.ijrobp.2006.06.043 17011458

[9] Hartl BA Ma HSW Hansen KS Perks J Kent MS Fragoso RC. The effect of radiation dose on the onset and progression of radiation-induced brain necrosis in the rat model. Int J Radiat Biol. (2017) 93:676–82. 10.1080/09553002.2017.1297902 28306402 PMC5751742

[10] Deng Z Huang H Wu X Wu M He G Guo J. Distinct expression of various angiogenesis factors in mice brain after whole-brain irradiation by X-ray. Neurochem Res. (2017) 42:625–33. 10.1007/s11064-016-2118-3 27885577

[11] Bálentová S Hnilicová P Kalenská D Murín P Hajtmanová E Lehotský J. Effect of whole-brain irradiation on the specific brain regions in a rat model: metabolic and histopathological changes. Neurotoxicology. (2017) 60:70–81. 10.1016/j.neuro.2017.03.005 28330762

[12] Rodgers SP Zawaski JA Sahnoune I Leasure JL Gaber MW. Radiation-induced growth retardation and microstructural and metabolite abnormalities in the hippocampus. Neural Plast. (2016) 2016:3259621. 10.1155/2016/3259621 27242931 PMC4875992

[13] Lawrie TA Gillespie D Dowswell T Evans J Erridge S Vale L Long-term neurocognitive and other side effects of radiotherapy, with or without chemotherapy, for glioma. Cochrane Database Syst Rev. (2019) 8:CD013047 10.1002/14651858.CD013047.pub2 31425631 PMC6699681

[14] Ungvari Z Tarantini S Hertelendy P Valcarcel-Ares MN Fülöp GA Logan S. Cerebromicrovascular dysfunction predicts cognitive decline and gait abnormalities in a mouse model of whole brain irradiation-induced accelerated brain senescence. Geroscience. (2017) 39:33–42. 10.1007/s11357-017-9964-z 28299642 PMC5352588

[15] Kleinberg L Sloan L Grossman S Lim M. Radiotherapy, lymphopenia, and host immune capacity in glioblastoma: a potentially actionable toxicity associated with reduced efficacy of radiotherapy. Neurosurgery. (2019) 85:441–53. 10.1093/neuros/nyz198 31232425

[16] Toraih EA El-Wazir A Abdallah HY Tantawy MA Fawzy MS. Deregulated microRNA signature following glioblastoma irradiation. Cancer Control. (2019) 26:1073274819847226. 10.1177/1073274819847226 31046428 PMC6501491

[17] Sultana N Sun C Katsube T Wang B. Biomarkers of brain damage induced by radiotherapy. Dose Response. (2020) 18:1559325820938279. 10.1177/1559325820938279 32694960 PMC7350401

[18] Zhang P Chen Y Zhu H Yan L Sun S Pei S. The effect of gamma-ray-induced central nervous system injury on peripheral immune response: an *in vitro* and *in vivo* study. Radiat. Res. (2019) 192:440–50. 10.1667/RR15378.1 31393823

[19] Raviraj R Nagaraja SS Selvakumar I Mohan S Nagarajan D. The epigenetics of brain tumors and its modulation during radiation: a review. Life Sci. (2020) 256:117974. 10.1016/j.lfs.2020.117974 32553924

[20] Fike JR Rosi S Limoli CL. Neural precursor cells and central nervous system radiation sensitivity. Semin Radiat Oncol. (2009) 19:122–32. 10.1016/j.semradonc.2008.12.003 19249650 PMC2683587

[21] Hide T Komohara Y. Oligodendrocyte progenitor cells in the tumor microenvironment. Adv Exp Med Biol. (2020) 1234:107–22. 10.1007/978-3-030-37184-5_8 32040858

[22] Hoelzinger DB Demuth T Berens ME. Autocrine factors that sustain glioma invasion and paracrine biology in the brain microenvironment. J Natl Cancer Inst. (2007) 99:1583–93. 10.1093/jnci/djm187 17971532

[23] Wade A Robinson AE Engler JR Petritsch C James CD Phillips JJ. Proteoglycans and their roles in brain cancer. FEBS J. (2013) 280:2399–417. 10.1111/febs.12109 23281850 PMC3644380

[24] Iozzo RV Sanderson RD. Proteoglycans in cancer biology, tumour microenvironment angiogenesis. J Cell Mol Med. (2011) 15:1013–31. 10.1111/j.1582-4934.2010.01236.x 21155971 PMC3633488

[25] Theocharis AD Karamanos NK Proteoglycans remodeling in cancer: underlying molecular mechanisms. Matrix Biol. (2019) 75–76:220–59. 10.1016/j.matbio.2017.10.008 29128506

[26] Vitale D Kumar Katakam S Greve B Jang B Oh ES Alaniz L. Proteoglycans and glycosaminoglycans as regulators of cancer stem cell function and therapeutic resistance. FEBS J. (2019) 286:2870–82. 10.1111/febs.14967 31230410

[27] Silver DJ Siebzehnrubl FA Schildts MJ Yachnis AT Smith GM Smith AA. Chondroitin sulfate proteoglycans potently inhibit invasion and serve as a central organizer of the brain tumor microenvironment. J Neurosci. (2013) 33:15603–17. 10.1523/JNEUROSCI.3004-12.2013 24068827 PMC3782629

[28] Kim Y Kang H Powathil G Kim H Trucu D Lee W. Role of extracellular matrix and microenvironment in regulation of tumor growth and LAR-mediated invasion in glioblastoma. PLoS One. (2018) 13:e0204865. 10.1371/journal.pone.0204865 30286133 PMC6171904

[29] Lee WH Warrington JP Sonntag WE Lee YW. Irradiation alters MMP-2/TIMP-2 system and collagen type IV degradation in brain. Int J Radiat Oncol Biol Phys. (2012) 82:1559–66. 10.1016/j.ijrobp.2010.12.032 22429332 PMC3309226

[30] Politko MO Prokaeva AI Pashkovskaya OA Kuper KE Zheravin AA Kliver EE. Single X-ray irradiation modulates proteoglycan expression in brain tissue: investigation using mouse model. Mol Biol Rep. (2020) 47:5657–63. 10.1007/s11033-020-05578-1 32514998

[31] Nicolson GL Custead SE Dulski KM Milas L. Effects of gamma irradiation on cultured rat and mouse microvessel endothelial cells: metastatic tumor cell adhesion, subendothelial matrix degradation, and secretion of tumor cell growth factors. Clin Exp Metastasis. (1991) 9:457–68. 10.1007/BF01785531 1914280

[32] Andrews RN Metheny-Barlow LJ Peiffer AM Hanbury DB Tooze JA Bourland JD. Cerebrovascular remodeling and neuroinflammation is a late effect of radiation-induced brain injury in non-human primates. Radiat Res. (2017) 187:599–611. 10.1667/RR14616.1 28398880 PMC5508216

[33] Andrews RN Caudell DL Metheny-Barlow LJ Peiffer AM Tooze JA Bourland JD. Fibronectin produced by cerebral endothelial and vascular smooth muscle cells contributes to perivascular extracellular matrix in late-delayed radiation-induced brain injury. Radiat Res. (2018) 190:361–73. 10.1667/RR14961.1 30016219 PMC6191839

[34] Fernström E Minta K Andreasson U Sandelius Å Wasling P Brinkmalm A Cerebrospinal fluid markers of extracellular matrix remodelling, synaptic plasticity neuroinflammation before after cranial radiotherapy. J Intern Med. (2018) 284:211–25. 10.1111/joim.12763 29664192

[35] Svendsen A Verhoeff JJ Immervoll H Brøgger JC Kmiecik J Poli A. Expression of the progenitor marker NG2/CSPG4 predicts poor survival and resistance to ionising radiation in glioblastoma. Acta Neuropathol. (2011) 122:495–510. 10.1007/s00401-011-0867-2 21863242 PMC3185228

[36] Pei J Park IH Ryu HH Li SY Li CH Lim SH. Sublethal dose of irradiation enhances invasion of malignant glioma cells through p53-MMP 2 pathway in U87MG mouse brain tumor model. Radiat Oncol. (2015) 10:164. 10.1186/s13014-015-0475-8 26245666 PMC4554349

[37] Yoo KC Suh Y An Y Lee HJ Jeong YJ Uddin N. Proinvasive extracellular matrix remodeling in tumor microenvironment in response to radiation. Oncogene. (2018) 37:3317–28. 10.1038/s41388-018-0199-y 29559744

[38] Shankar A Kumar S Iskander AS Varma NR Janic B deCarvalho A. Subcurative radiation significantly increases cell proliferation, invasion, and migration of primary glioblastoma multiforme *in vivo*. Chin J Cancer. (2014) 33:148–58. 10.5732/cjc.013.10095 24016393 PMC3966215

[39] Zhu C Mao X Zhao H. The circ_VCAN with radioresistance contributes to the carcinogenesis of glioma by regulating microRNA-1183. Medicine (Baltimore). (2020) 99:e19171. 10.1097/MD.0000000000019171 32080097 PMC7034728

[40] Suhovskih AV Kazanskaya GM Volkov AM Tsidulko AY Aidagulova SV Grigorieva EV. Chemoradiotherapy increases intratumor heterogeneity of HPSE expression in the relapsed glioblastoma tumors. Int J Mol Sci. (2020) 21:E1301. 10.3390/ijms21041301 32075104 PMC7073003

[41] Hinks GL Chari DM O'Leary MT Zhao C Keirstead HS Blakemore WF. Depletion of endogenous oligodendrocyte progenitors rather than increased availability of survival factors is a likely explanation for enhanced survival of transplanted oligodendrocyte progenitors in X-irradiated compared to normal CNS. Neuropathol Appl Neurobiol. (2001) 27:59–67. 10.1046/j.0305-1846.2001.00303.x 11299003

[42] Irvine KA Blakemore WF. A different regional response by mouse oligodendrocyte progenitor cells (OPCs) to high-dose X-irradiation has consequences for repopulating OPC-depleted normal tissue. Eur J Neurosci. (2007) 25:417–24. 10.1111/j.1460-9568.2007.05313.x 17284182

[43] Miyata S Kitagawa H. Formation and remodeling of the brain extracellular matrix in neural plasticity: roles of chondroitin sulfate and hyaluronan. Biochim Biophys Acta Gen Subj. (2017) 1861:2420–34. 10.1016/j.bbagen.2017.06.010 28625420

[44] Xiong A Kundu S Forsberg-Nilsson K. Heparan sulfate in the regulation of neural differentiation and glioma development. FEBS J. (2014) 281:4993–5008. 10.1111/febs.13097 25284049

[45] Xiong A Spyrou A Forsberg-Nilsson K. Involvement of heparan sulfate and heparanase in neural development and pathogenesis of brain tumors. Adv Exp Med Biol. (2020) 1221:365–403. 10.1007/978-3-030-34521-1_14 32274718

[46] Saito T Sugiyama K Hama S Yamasaki F Takayasu T Nosaka R. High expression of glypican-1 predicts dissemination and poor prognosis in glioblastomas. World Neurosurg. (2017) 105:282–8. 10.1016/j.wneu.2017.05.165 28602885

[47] Krishnaswamy VR Benbenishty A Blinder P Sagi I. Demystifying the extracellular matrix and its proteolytic remodeling in the brain: structural and functional insights. Cell Mol Life Sci. (2019) 76:3229–48. 10.1007/s00018-019-03182-6 31197404 PMC11105229

[48] De Luca C Papa M. Matrix Metalloproteinases, neural extracellular matrix, and central nervous system pathology. Prog Mol Biol Transl Sci. (2017) 148:167–202. 10.1016/bs.pmbts.2017.04.002 28662822

[49] Ilan N Bhattacharya U Barash U Boyango I Yanku Y Gross-Cohen M. Heparanase-the message comes in different flavors. Adv Exp Med Biol. (2020) 1221:253–83. 10.1007/978-3-030-34521-1_9 32274713

[50] Gupta K Burns TC. Radiation-induced alterations in the recurrent glioblastoma microenvironment: therapeutic implications. Front Oncol. (2018) 8:503. 10.3389/fonc.2018.00503 30467536 PMC6236021

[51] Zhao X Li C Liu L Zou H Li K. Identification of aberrantly expressed genes in murine glioblastoma during radiotherapy via bioinformatic data mining. Onco Targets Ther. (2020) 13:3839–51. 10.2147/OTT.S247794 32440151 PMC7212782

[52] Han W Umekawa T Zhou K Zhang XM Ohshima M Dominguez CA. Cranial irradiation induces transient microglia accumulation, followed by long-lasting inflammation and loss of microglia. Oncotarget. (2016) 7:82305–23. 10.18632/oncotarget.12929 27793054 PMC5347693

[53] Yang N Gao X Qu X Zhang R Tong F Cai Q. PIDD mediates radiation-induced microglia activation. Radiat Res. (2016) 186:345–59. 10.1667/RR14374.1 27643878

[54] Seo YS Ko IO Park H Jeong YJ Park JA Kim KS. Radiation-induced changes in tumor vessels and microenvironment contribute to therapeutic resistance in glioblastoma. Front Oncol. (2019) 9:1259. 10.3389/fonc.2019.01259 31803626 PMC6873882

